# Supplementary material for: First body of evidence suggesting a role of a tankyrase-binding motif (TBM) of vinculin (VCL) in epithelial cells
Source: PeerJ. 2021 May 27;9:e11442. doi: 10.7717/peerj.11442 (PMC8164839; doi:10.7717/peerj.11442)
Supplement: Supplemental Information 1 [file peerj-09-11442-s001.docx]

**First evidences suggesting a role of a Tankyrase-Binding Motif (TBM) of Vinculin (VCL) in epithelial cells.**

**Supplemental material**

Contents

[Supplemental_Materials and Methods 1](#_Toc69460004)

[1. PAR-VCL and VCL-PAR indirect ICF and FRET assay 1](#_Toc69460005)

[2. Obtainment of MCF-7/Knock cells: targeting of MCF-7 endogenous hVCL (on exon 3) with CRISPR/Cas9 ribonucleoproteins. 3](#_Toc69460006)

[3. MCF-7 /Knock cells cloning attempt by the arrays method 4](#_Toc69460007)

[4. Analysis of MCF-7/Knock hVCL CRISPR-Cas target locus in exon 3 (633bp) 4](#_Toc69460008)

[5. Transfection of MCF-7/Knock monolayers or suspensions with Tol2-GgVCL or Tol2-GgVCL/*TBM 5](#_Toc69460009)

[Supplemental_Figure Legends 5](#_Toc69460010)

[Supplemental Figure S1. TNKS antibody controls. 5](#_Toc69460011)

[Supplemental Figure S2. Affinity precipitation of PARylated proteins with Macrodomain resin was followed by WB to detect VCL and PARP-1. 5](#_Toc69460012)

[Supplemental Figure S3. Colocalization of PAR and VCL in the epithelial belt and colocalization loss in a EMT model. 5](#_Toc69460013)

[Supplemental Figure S4. FRET VCL and PAR colocalization within 50 nm 5](#_Toc69460014)

[Supplemental Figure S5. MCF-7 /”Knock-out” cells: gRNA designs and phenotype (days-1 month) 6](#_Toc69460015)

[Supplemental Figure S6. MCF-7 /”Knock-out” cells: gRNA designs and phenotype (1 to 3 months) 6](#_Toc69460016)

[Supplemental Figure S7. Transfection of “Knocked” cells with either Tol2-GgVCL or Tol2-GgVCL/*TBM. 6](#_Toc69460017)

[Supplemental Figure S8. Cell shape index changes induced by “KO” or VCL/*TBM 7](#_Toc69460018)

[Supplemental References (FRET) 7](#_Toc69460019)

# Supplemental_Materials and Methods

## PAR-VCL and VCL-PAR indirect ICF and FRET assay

In order to do indirect ICF and FRET, 200.000 cells/well were seeded on 12 mm diameter coverslips inside 24-well plates. Primary antibodies used were 1:100 rabbit anti-PAR (BD) and 1:200 mouse anti-vinculin. Then, two sets of secondary antibodies combinations were used (all 1:1000) to analyze putative FRET from PAR to VCL and vice versa. Set A: from PAR to VCL_goat anti-rabbit Alexa 488 (A11034) & goat anti-mouse Alexa Fluor 546 (A11030); Set B: from VCL to PAR_goat anti-mouse Alexa 488 (A11029) & goat anti-rabbit Alexa 546 (A11035). As FRET signal is dependent not only on the distance among the fluorophores but also on angles, even if fluorophores are near enough (within 100 Angstroms or 10 nm), FRET may not occur.

Images were taken at 60 x, zoom 2, accumulated from 6 reads, at OLYMPUS FV. The register was always taken in the same order (f, e, g, c, d, b, a, then the control without primary antibodies), being: a: Donor sample, donor channel; b: Donor sample, FRET channel; c: acceptor sample, FRET channel; d: acceptor sample, acceptor channel; e: Combined sample, donor channel; f: Combined sample, FRET channel; g: Combined sample, acceptor channel. Images were converted to RGB using the FV control plugin and then to 8-bit tiff. The control without primary antibodies was used as in any ICF experiment to decide the maximum laser and PMT allowed values to assure that the register is signal. On the other hand, saturatin was avoided. Laser 488 was set to 10% and laser 543 to 25 %, with both PMT to 500 V. Offset was set to 0 and gain to 1. The settings were maintained constant during the whole register.

The general recommendation to do FRET is to prepare 3 biological samples (besides the control without any primary antibody): one harboring only the donor primary antibody (sample D), one harboring the acceptor primary antibody (sample A) and one with both antibodies to analyze FRET (FRET sample). All the samples carry both secondary antibodies. Then, register each sample in four channels: donor channel (green, as usual; laser 488 and register in 510-530 nm); acceptor channel (red, as usual; laser 543 and register at 600-630 nm), FRET channel (laser 488 and register at 600-630nm) and the control or reverse channel (laser 543 and register at 520-530 nm). The latter is expected to be zero from a physical point of view, since energy is lost as heat but not gained, and lower wavelength means higher energy. Thus, such channel, which would only register the system background noise, was skipped.

Using the donor samples, ImageJ “FRET and colocalization analyser” plugin computes intensities pixel by pixel and generates a linear regression equation in which the slope corresponds to the donor spillover.

$$Spillover D= \frac{FRET channel (image b)}{D channel (image a)}$$

Analogously, using the acceptor samples, it computes the acceptor spillover

$$Spillover A=\frac{FRET channel (image c)}{A channel (image d)}$$

Then, the FRET sample is imaged in donor channel, FRET channel and acceptor channel. The expected total spillover in each pixel is:

$$Spillover T=(Spillover D x D channel image e) + (Spillover A x A channel image g)$$

Then FRET is computed pixel per pixel according to Youvan et al. (2003) as the increase in FRET channel over the expected background from spillover:

$$corrected FRET =FRET channel \left( image f \right)-Spillover T$$

ImageJ “FRET and colocalization analyser” plugin provides additional information to check the quality of the data obtained.

A regression graph (FRET channel intensity vs donor channel intensity for each pixel) and a regression control image indicate the pixels in the image that are more or less intense than expected by each Spillover coefficient. Red: above the expected; blue: below the expected. Red points will be undercorrected and thus will give false positive FRET signals. Thus, it should be checked that the structures of interest are not covered by red points in these control images, and if you later get FRET on structures that are blue in these control images, it means that FRET is high enough to surpass the negative bias.

The FRET index image displays the intensities of the acceptor emission due to FRET at each point. The color corresponds to the “fire” Look-Ot-Table, such that blue is no FRET and red is high FRET. Finally, false-positive points with FRET signal in the absence of colocalized donor and acceptor can be excluded, giving rise to the “colocalized FRET index” image. In the colocalization diagram and FRET graph, the color of the points represents the mean of the indices of FRET calculated for the correponding pixels.

## Obtainment of MCF-7/Knock cells: targeting of MCF-7 endogenous hVCL (on exon 3) with CRISPR/Cas9 ribonucleoproteins.

The hVLC-gene KO kit was a kind gift from Synthego. Following the kit instructions and working in RNAse-free conditions, MCF-7 cells were transfected with ribonucleoproteins Cas9-VCL sgRNAs 1 to 4 (see guiding sequences in Table 3) alone or combined, either (i) in a classical Gene Pulser electroporator (0.45 KV, 250 µF, 1000 Ὢ; Ƭ≈10) using 10^6^ cells in a cold buffer based on Gene Pulser Patent Buffer (Gppb; 10 mM Hepes, 160 mM NaCl, 50 mM mannitol, 3% BSA) and immediate transference of electroporated cells to a 50 µL volume containing the ribonucleoparticles, or (ii) in a Lonza Nucleofector (P-020) using 3 10^5^ cells in a total volume of 100 µL 1M buffer (5 mM KCl, 15 mM MgCl2, 120 mM Na_2_HPO_4_/ NaH_2_PO_4_ pH 7.2, 50 mM mannitol). Electroporation/nucleofection controls were always done in parallel.

| sgRNA1 (fw strand; PAM on rev); #VCL74070730 | C*U*C*GAGCAGGCACUGAGUAA + SmEZscaff |
| --- | --- |
| sgRNA2 (fw strand; PAM on rev); #VCL74070703 | A*C*U*GAAGCAUCUGAGCUGCC + SmEZscaff |
| sgRNA3 (reverse strand: PAM on FW); #VCL74070696 | U*G*C*UUGCACCAAGCUUGUCC + SmEZscaff |
| sgRNA4 (fw strand; PAM on rev); #VCL74070693 | C*U*G*AGCUGCCUGGACAAGCU + SmEZscaff |

Table S1. **Guide RNAs sequences.** Each synthetic single-guide RNA (sgRNA) comprises a sequence complementary to the target (CRISPR RNA or guide sequence, here depicted) + a helper and Synthego modified EZ scaffold (SmEZscaff) sequence (transactivating CRISPR RNA). For Cas9 from *Streptococcus pyrogenes*, the short protospacer adjacent motif (PAM) that has to be downstream the target DNA is 5’-NGG-3’, where N is any nucleotide .

MCF-7 cells subjected to electroporation or nucleofection of ribonucleoproteins Cas9/ VCL-targeting sgRNAs were called MCF-7/Knock cells and were seeded in DMEM+20% FBS in 24 well plates for subsequent follow-up.

## MCF-7 /Knock cells cloning attempt by the arrays method

Thirty-three days after electroporation with Cas9 + sgRNA (1+3) RNPs, 4 10^3^ cells were seeded in the A1 well of a 96 wells plate in 200 µL complete medium. Serial 1:2 dilutions were done first top-bottom in column 1 and then from left to right in each row. Individual cells did not survive. Thus, we could not obtain pure clones. We obtained what we called semi-clones in 8 of the wells, which were seeded on coverglasses in 24 well plates and subjected to ICF 72 days later (33 + 72 = 105 days after electroporation).

## Analysis of MCF-7/Knock hVCL CRISPR-Cas target locus in exon 3 (633bp)

To extract genomic DNA, 5 x 10^5^ MCF-7 control or MCF-7/Knock cells grown in 24-well plates were shaken in 100 µL lysis buffer (100 mM Tris pH 8, 5mM EDTA, 0.2% SDS, and 200 mM NaCl) 5 min at room temperature. Each extract was collected in an eppendorf. After chloroform (100 µL) addition and mixing by inversion, it was centrifuged at 5000 RPM 5 min. The aqueus phase was collected, 200 µL chloroform was added, and after mixing, centrifuging and rescuing the aqueus phase again, DNA was precipitated with isopropanol at 13000 RMP 20 min at 4°C. Then genomic DNA was quantified in a Nanodrop and used as template to amplify the CRISPR-Cas target locus in exon 3 using custom-designed primer pair 9 (PP9):

| **PP9** (#10336022: P180917-006: A03 & A04) **for** e**xon3 (CRISPR-Cas9 targets) flanking genomic locus** | Fwd: GCATTGCAGAGATAGAGCAACC |
| --- | --- |
|  | Rev: TGGGTGGACAACTAGCAAACA |

The PCR reaction mix contained Invitrogen or home-made PCR buffer (10x: 1M Tris-HCl pH 8.4, 400 mM NaCl, 100 mM spermidine, 15 mM MgCl_2_), 0.2 mM each dNTP, 0.5 µM each primer, plus 1 U Invitrogen Taq DNA pol and 50 ng MCF-7 DNA per reaction. A negative control was always included.

PP9 PCR products (633 pb) from MCF-7 control cells (5 µL, unpurified, 200 ng/mL) and MCF-7/Knock cells (10 µL, purified, 25 ng/mL) were sent to Macrogen for Sanger sequencing in order to check whether VCL gene had been knocked out in “knocked” cells (obtained with combined sgRNA1 and sgRNA3).

## Transfection of MCF-7/Knock monolayers or suspensions with Tol2-GgVCL or Tol2-GgVCL/*TBM

1 μg Pmax GFP plasmid (SIZE~3KB) from Amaxa Mouse/Rat Hepatocyte Nucleofector kit was used as a positive control to evaluate transfection efficiency of a DNA plasmid. Gene Pulser electroporation was not effective to transfect plasmids to MCF-7 cells (for some reason, plasmid entry seems to be more difficult than RNPs entry). Thus, either Lonza Nucleofector (P-020) was used to transfect MCF-7/Knock cells in suspension as described above or FUGENE transfection reaction: DNA (3:1) was used to transfect MCF-7/Knock undisrupted monolayers. Cells were watched under epifluorescence and phase contrast or subjected to ICF at the indicated times.

# Supplemental_Figure Legends

## Supplemental Figure S1. TNKS antibody controls.

Left column: controls without primary antibody. Right column: anti-TNKS ICF was performed with different antibodies (GeneTex rabbit anti-TNKS-Nt or Novex mouse anti-hTNKS, catalytic region (1106-1325)) and under different fixation protocols, namely 4 % PFA or a combination of PFA and glutaraldehyde (bottom).

## Supplemental Figure S2. Affinity precipitation of PARylated proteins with Macrodomain resin was followed by WB to detect VCL and PARP-1.

**(A**) Cells were seeded in the absence or presence of TNKSi G007LK and lysed 5 h later. (**B**) Cells were grown without or with TNKS+PARP-1/2/3 inhibitor XAV939 until confluence and then lysed. Arrows point at the VCL species that was enriched in the AP fraction in the absence of TNKSi (next to white asterisks).

## Supplemental Figure S3. Colocalization of PAR and VCL in the epithelial belt and colocalization loss in a EMT model.

VCL (*red)*, PAR (*green)* and colocalizing pixels (*yellow)*. (**A-C**) Vero cells, 100x; (**D**): Vero cell monolayer overview; (**E - H**): correspondent masks; (**I - L**): masks products highlighting colocalization at the epithelial belt. (**M**, **N**): NMuMG cells. (**M**): untreated or **(N**): after EMT induction by TGF-β.

## Supplemental Figure S4. FRET VCL and PAR colocalization within 50 nm

FRET was done after indirect ICF using secondary antibodies bound to Alexa 488 as donor fluorophores and secondary antibodies bound to Alexa 546 as receptors. (**A - F**) PAR -bound Alexa 488 and VCL- bound Alexa 546 and (**G - L**) *vice versa*. Then, FRET was evaluated using the ImageJ Fret and Colocalization plugin, allowing the localization of the subcellular structures where vinculin and PAR colocalized within about 50 nm resolution (10 nm FRET + 10 nm each primary and each secondary antibody, see König *et al* (2006).

(**A, G**) Relative intensity graphs. (**B, H**) Donor bleed through or spillover image. Red points are more intense than expected by spillover coefficient. Thus, it is important to check that the structures of interest are not in red. The opposite is true for blue points. (**C, I**) Analogous acceptor bleed through. (**D, J**) Sample confocal images. (**E, K**) FRET index represents the intensities of the acceptor emission due to FRET. Blue is no FRET, violet/red is FRET. (**F, L**) False positive points with FRET signal in the absence of colocalized donor and acceptor can be excluded, giving this Colocalized FRET index images. Again, violet points represent FRET.

## Supplemental Figure S5. MCF-7 /”Knock-out” cells: gRNA designs and phenotype (days-1 month)

**(A)** Each synthetic single-guide RNA (sgRNA) comprises a sequence complementary to the target (CRISPR RNA or guide sequence, here depicted) + a helper and scaffold sequence (transactivating CRISPR RNA) (not-shown). For Cas9 from *Streptococcus pyrogenes*, the short protospacer adjacent motif (PAM) that has to be downstream the target DNA is 5’-NGG-3’, where N is any nucleotide. The 20 nt-sequences complementary to the gRNA were on the FW strand for gRNA 1, 2 and 4 (yellow rectangles) and on the REV strand for gRNA 3. As all gRNAs were located on the third VCL exon, a primer pair (PP9 FW and PP9 REV) was designed to amplify such region for subsequent checking of the changes obtained for this sequence. (**B - E**) MCF-7 cells were nucleofected in the absence or presence of RNPs of Cas9 + the indicated sgRNAs and photographed 8 days later. (**B**) Nucleofection control; (**C**) sgRNA2; (**D, E**) sgRNA4; (**F-I**) MCF-7 cells were electroporated in the absence or presence of RNPs of Cas9 + the indicated sgRNAs and photographed 8 days later (**F**) Electroporation control; (**G**) sgRNA1; (**H**) sgRNA 3; (**I**) combined sgRNA 1 + sgRNA3. **(J - Q)** ICF with anti-VCL antibody (*green*) and DAPI counterstain (*blue*) of cells fixed 1 month later. (**J, N**): electroporation control, (**K,O**): sgRNA1, (**L,P**): sgRNA3, (**M,O**): sgRNa1 + sgRNA3. In electroporation control and sgRNA3, apical and basal regions were still distinguished (the insets represent VCL in basal region). In flattened cells, it was not possible to define apical vs basal regions and there is no inset.

## Supplemental Figure S6. MCF-7 /”Knock-out” cells: gRNA designs and phenotype (1 to 3 months)

Cells electroporated with sgRNA1 + sgRNA3 were followed and subjected to ICF. Here are more examples of microscopic fields: (**A - D**) one month and (**E - H**) 3 months after electroporation. These cells transfected with RNPs to *knock-out* VCL were named MCF-7/”Knock” cells. They were characterized by extremely slow cell cycling, no survival as single cells, cell flattening, and relatively low but variable VCL expression

## Supplemental Figure S7. Transfection of “Knocked” cells with either Tol2-GgVCL or Tol2-GgVCL/*TBM.

Cell monolayer was transfected with the plasmids of interest using FUGENE (**A - J**) while cell suspensions were nucleofected (**K - R**). Fixation was done 48 h later. GFP (*yellow*), VCL (*green*) and F-actin (*red*) were detected by ICF. (**A, F**) Monolayer overview, merged channels; (**B - E** & **G - J**): enlarged view of two Tol2-VCL/*TBM transfected cells. (**B**) merge, (**C**): GFP, (**D**): VCL+GFP, (**E**): VLC+F-actin+ drawn contour, (**G**): merge, (**H**): GFP, (**I**):VCL+GFP, (**J**): VLC+F-actin+ drawn contour. (**K - R**) Successful transfection of neighbor cells was achieved (**K - N**): with Tol2-GgVCL/*TBM; (**O - R**): with Tol2-GgVCL. (**K, O**): F-actin, (**L, P**): VCL, (**M,Q**): GFP, (**N,R**): DAPI. Bar: 25 µm

## Supplemental Figure S8. Cell shape index changes induced by “KO” or VCL/*TBM

# Supplemental References (FRET)

Konig, P.; Krasteva, G.; Tag, C.; Konig, I.R.; Arens, C.; Kummer, W. FRET-CLSM and double-labeling indirect immunofluorescence to detect close association of proteins in tissue sections. *Laboratory investigation; a journal of technical methods and pathology* **2006**, *86*, 853–864, doi:10.1038/labinvest.3700443

Youvan, D.C.; Silva, C.M.; Diagnostics, A.C.; Coleman, W.J.; Yang, M. Calibration of Fluorescence Resonance Energy Transfer in Microscopy Using Genetically Engineered GFP Derivatives on Nickel Chelating Beads Calibration of Fluorescence Resonance Energy Transfer in Microscopy Using Genetically Engineered GFP Derivatives on . **2003**.
