# Supplementary figures and images for: First body of evidence suggesting a role of a tankyrase-binding motif (TBM) of vinculin (VCL) in epithelial cells
Source: PeerJ. 2021 May 27;9:e11442. doi: 10.7717/peerj.11442 (PMC8164839; doi:10.7717/peerj.11442)

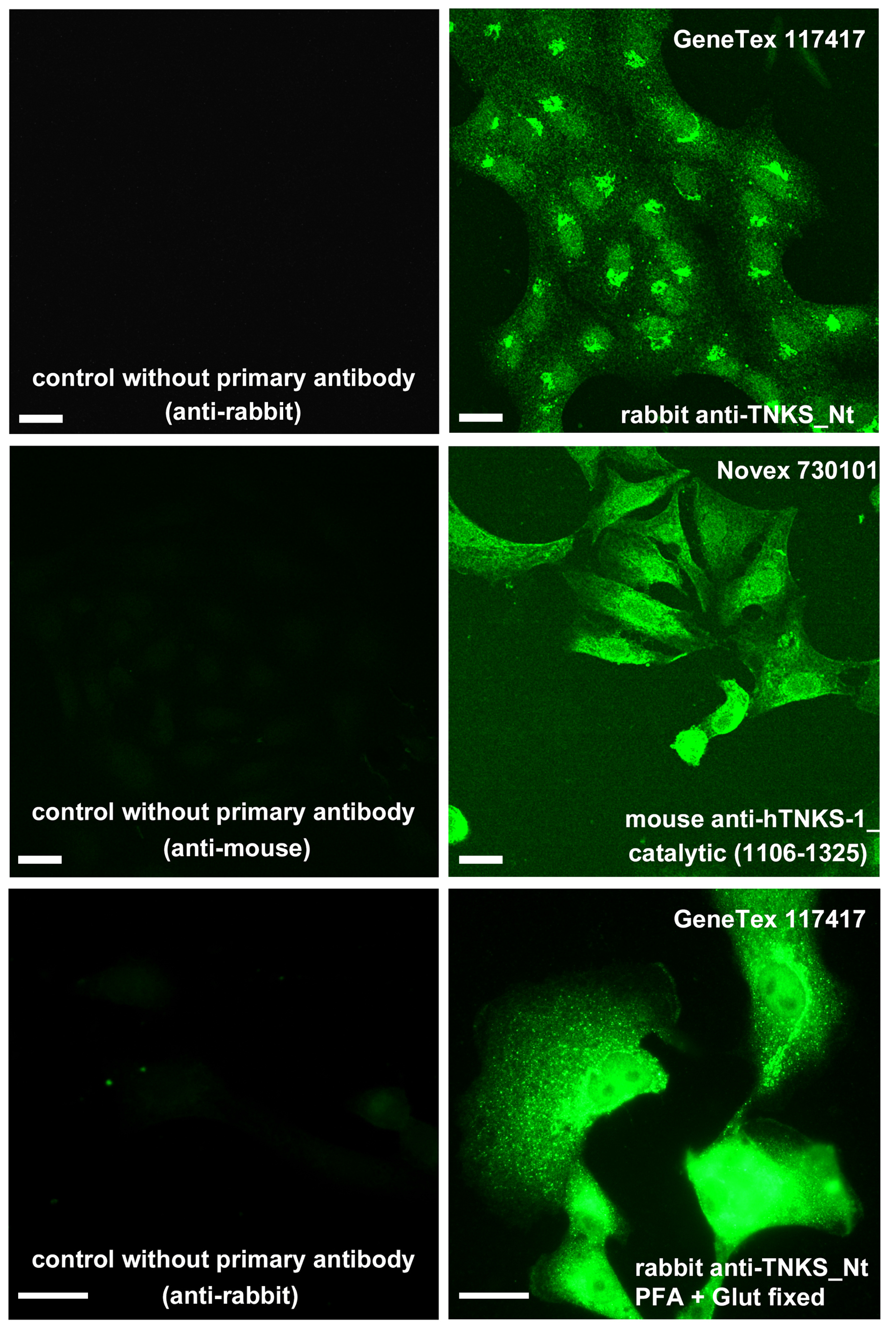

Supplement: Supplemental Information 2 — Left column: controls without primary antibody. Right column: anti-TNKS ICF was performed with different antibodies (GeneTex rabbit anti-TNKS-Nt or Novex mouse anti-hTNKS, catalytic region (1106-1325)) and under different fixation protocols, namely 4 % PFA or a combination of PFA and glutaraldehyde (bottom). [file peerj-09-11442-s002.png]

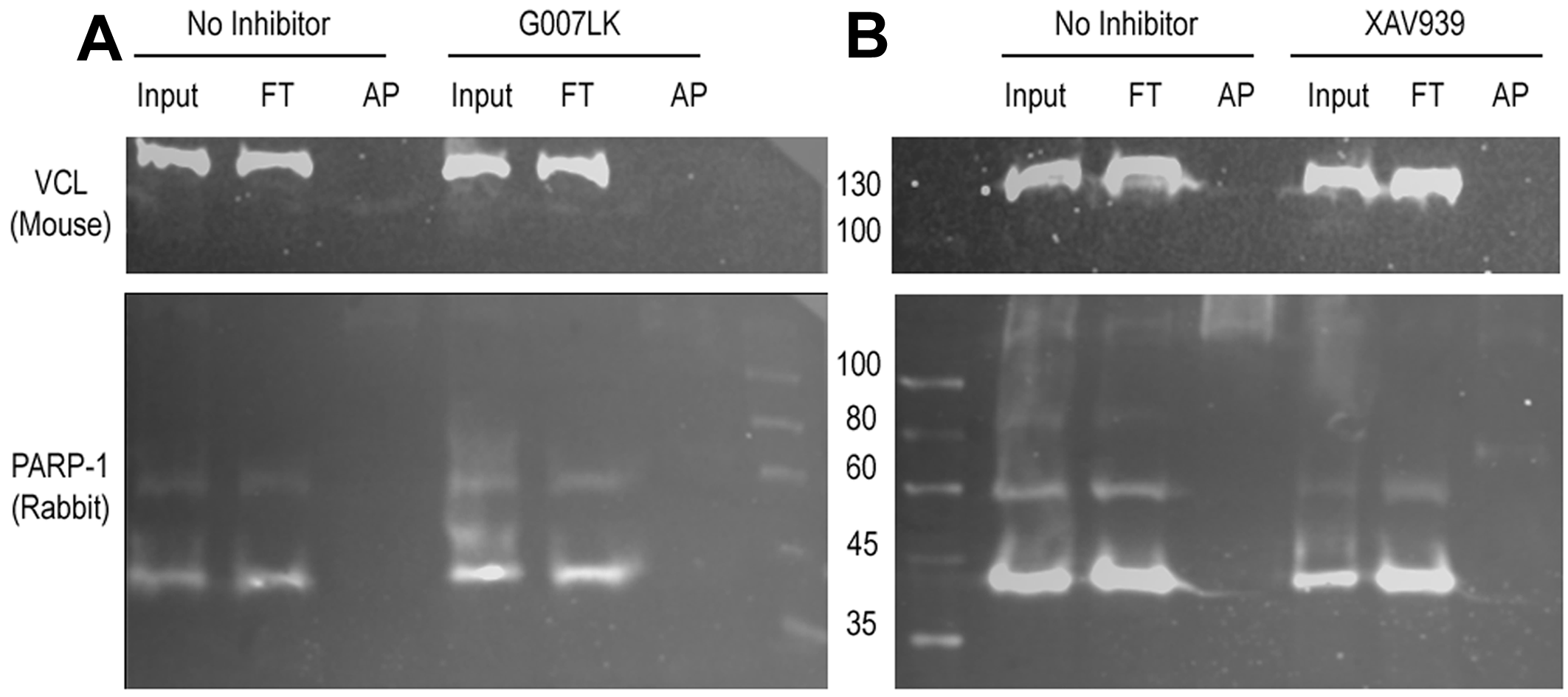

Supplement: Supplemental Information 3 — Affinity precipitation of PARylated proteins with Macro resin and WB to detect VCL and PARP-1. (A) Cells were seeded in the absence or presence of TNKSi G007LK and lysed 5 h later. (B) Cells were grown until confluency without or with TNKS+PARP-1/2/3 inhibitor XAV939 and lysed. Arrows point at the VCL species that was enriched in the AP fraction in the absence of TNKSi (next to white asterisks). [file peerj-09-11442-s003.png]

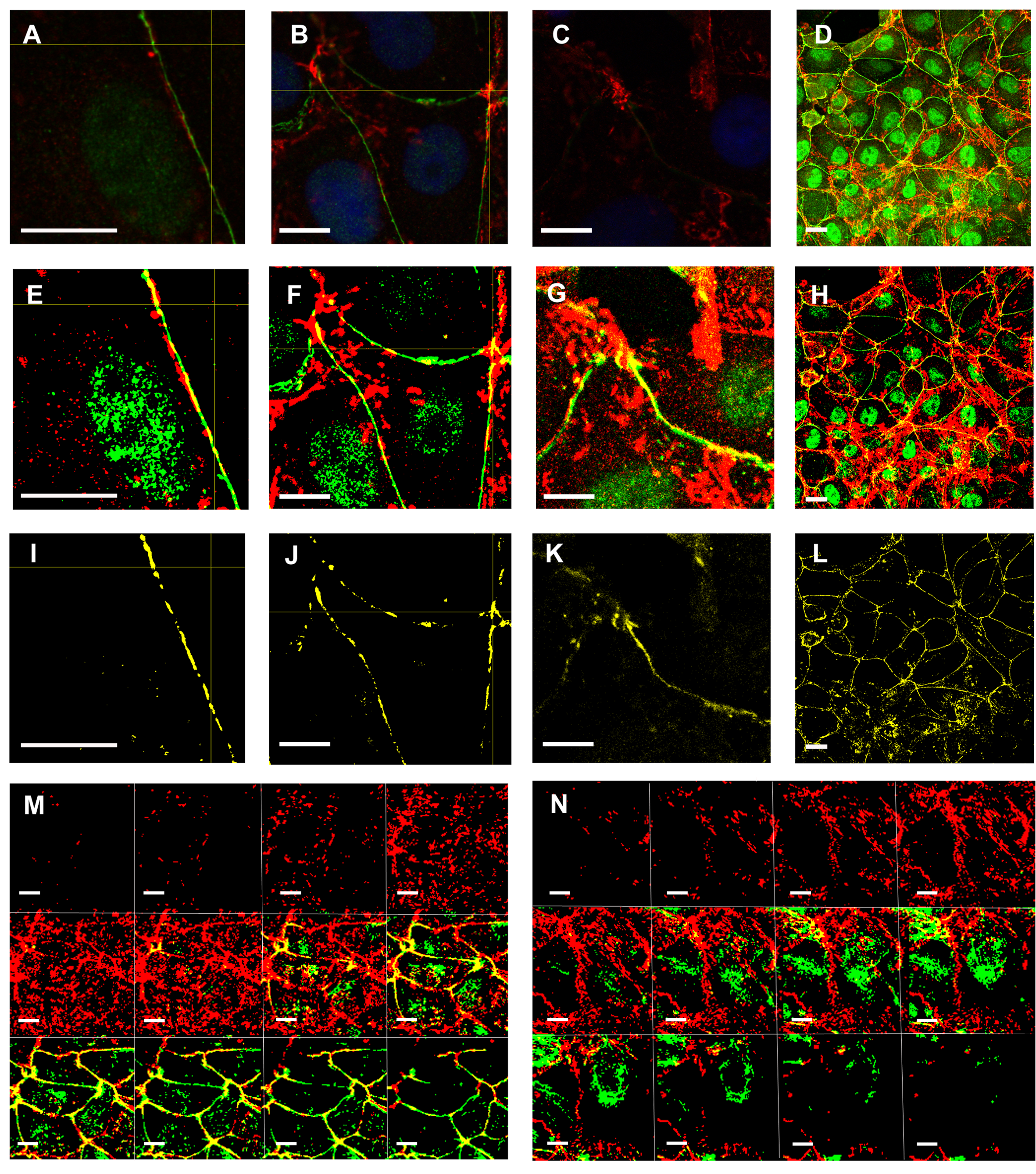

Supplement: Supplemental Information 4 — VCL (red), PAR (green) and colocalizing pixels (yellow). (A-C) Vero cells, 100x; (D): Vero cell monolayer overview; (E- H): correspondent masks; (I - L): masks products highlighting colocalization at the epithelial belt. (M, N): NMuMG cells. (M): untreated or (N): after EMT induction by TGF-β [file peerj-09-11442-s004.png]

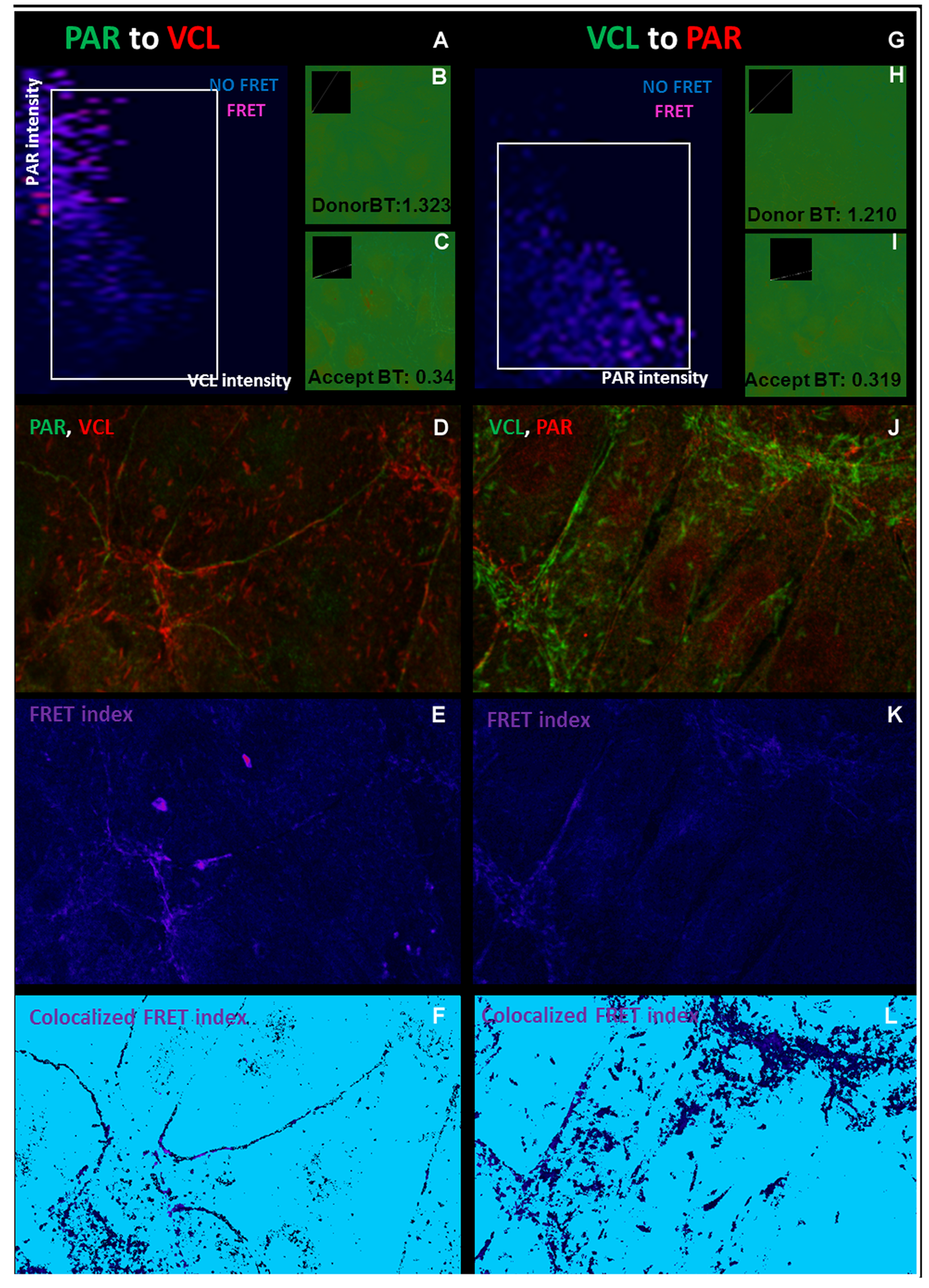

Supplement: Supplemental Information 5 — FRET was done after indirect ICF using secondary antibodies bound to Alexa 488 as donor fluorophores and secondary antibodies bound to Alexa 546 as receptors. (A – F) PAR -bound Alexa 488 and VCL- bound Alexa 546 and (G - L) viceversa. Then, FRET was evaluated using the ImageJ Fret and Colocalization.plugin, allowing the localization of the subcellular structures where vinculin and PAR colocalized within about 50 nm resolution (10 nm FRET + 10 nm each primary and each secondary antibody, see König et al 2006) (Kwan et al., 2007). (A, G) Relative intensity graphs. (B, H) Donor bleedthrough or spillover image. Red points are more intense than expected by spillover coefficient, will be undercorrected and may give false positive FRET. Thus, it is important to check that the structures of interest are not in red. The opposite is true for blue points. (C, I) Analogous acceptor bleedthrough (D, J) Sample confocal images (E, K) FRET index represents the intensities of the acceptor emission due to FRET. Blue is no FRET, violet/red is FRET (F, L) False positive points with FRET signal in the absence of colocalized donor and acceptor can be excluded, giving this Colocalized FRET index images. Again, violet points represent FRET. [file peerj-09-11442-s005.png]

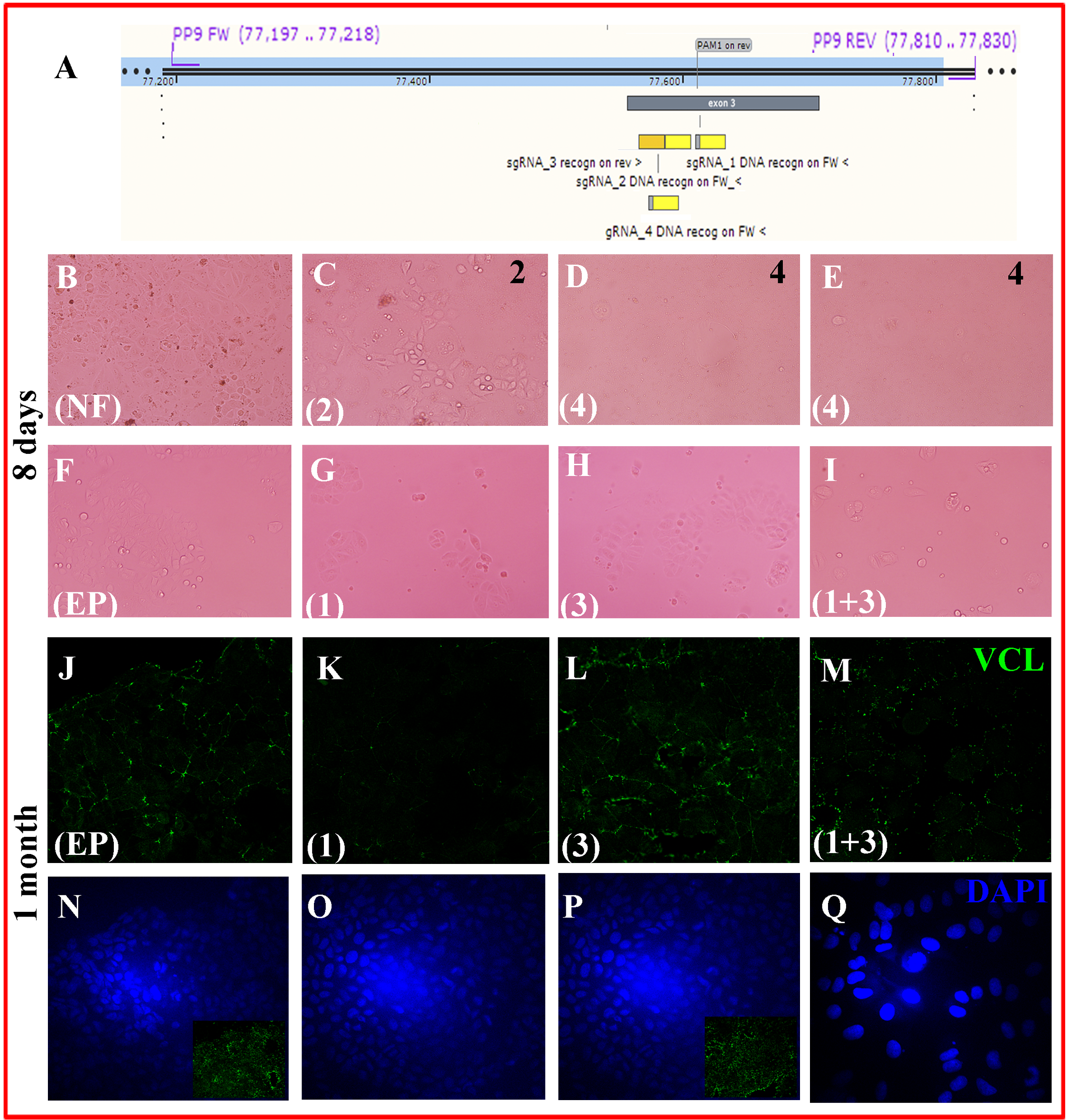

Supplement: Supplemental Information 6 — (A) Each synthetic single-guide RNA (sgRNA) comprises a sequence complementary to the target (CRISPR RNA or guide sequence, here depicted) + a helper and scaffold sequence (transactivating CRISPR RNA) (not-shown). For Cas9 from Streptococcus pyrogenes, the short protospacer adjacent motif (PAM) that has to be downstream the target DNA is 5’-NGG-3’, where N is any nucleotide. The 20 nt-sequences complementary to the gRNA were on the FW strand for gRNA 1, 2 and 4 (yellow rectangles) and on the REV strand for gRNA 3. As all gRNAs were located on the third VCL exon, a primer pair (PP9 FW and PP9 REV) was designed to amplify such region for subsequent checking of the changes obtained for this sequence. (B-E) MCF-7 cells were nucleofected in the absence or presence of RNPs of Cas9 + the indicated sgRNAs and photographed 8 days later. (B) Nucleofection control; (C) sgRNA2 (D, E) sgRNA4. (F-I) MCF-7 cells were electroporated in the absence or presence of RNPs of Cas9 + the indicated sgRNAs and photographed 8 days later (F) Electroporation control; (G) sgRNA1; (H) sgRNA 3; (I) combined sgRNA 1+ sgRNA3. <!--[if !supportLists]-->(B) <!--[endif]--> (J-Q) ICF with anti-VCL antibody (green) and DAPI counterstain (blue) of cells fixed 1 month later. (J, N): electroporation control, (K,O): sgRNA1, (L,P): sgRNA3, (M,O): sgRNa 1+sgRNA3.In electroporation control and sgRNA3, apical and basal regions were still distinguished (the insets represent VCL in basal region). In flattened cells, it was not possible to define apical vs basal regions and there is no inset. [file peerj-09-11442-s006.png]

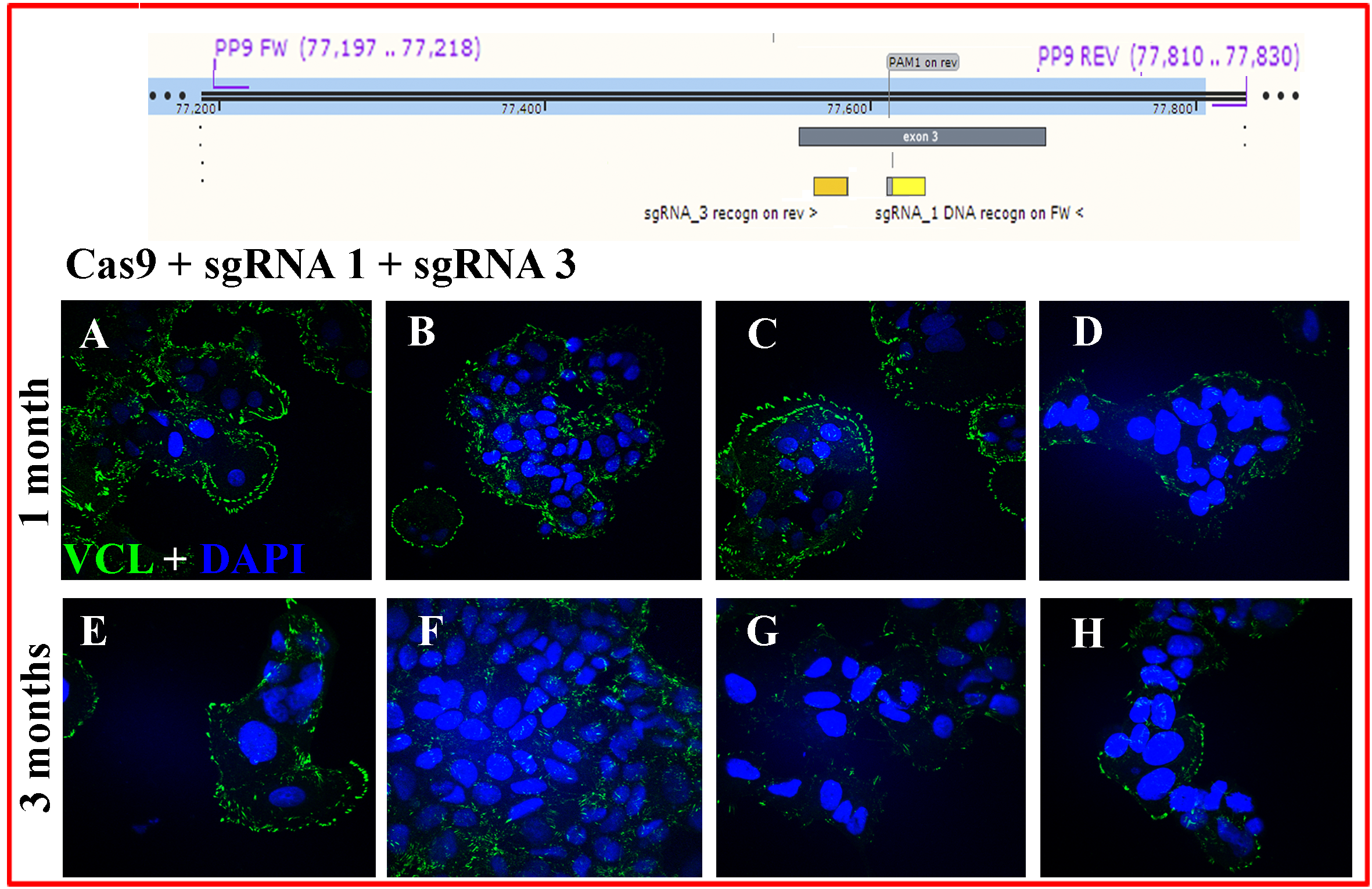

Supplement: Supplemental Information 7 — Cells electroporated with sgRNA1 + sgRNA3 were followed and subjected to ICF. Here are more examples of microscopic fields: (A-D) one month and (E-H) 3 months after electroporation. These cells transfected with RNPs to knock-out VCL were named MCF-7/”Knock” cells. They were characterized by extremely slow cell cycling, no survival as single cells, cell flattening, and relatively low but variable VCL expression [file peerj-09-11442-s007.png]

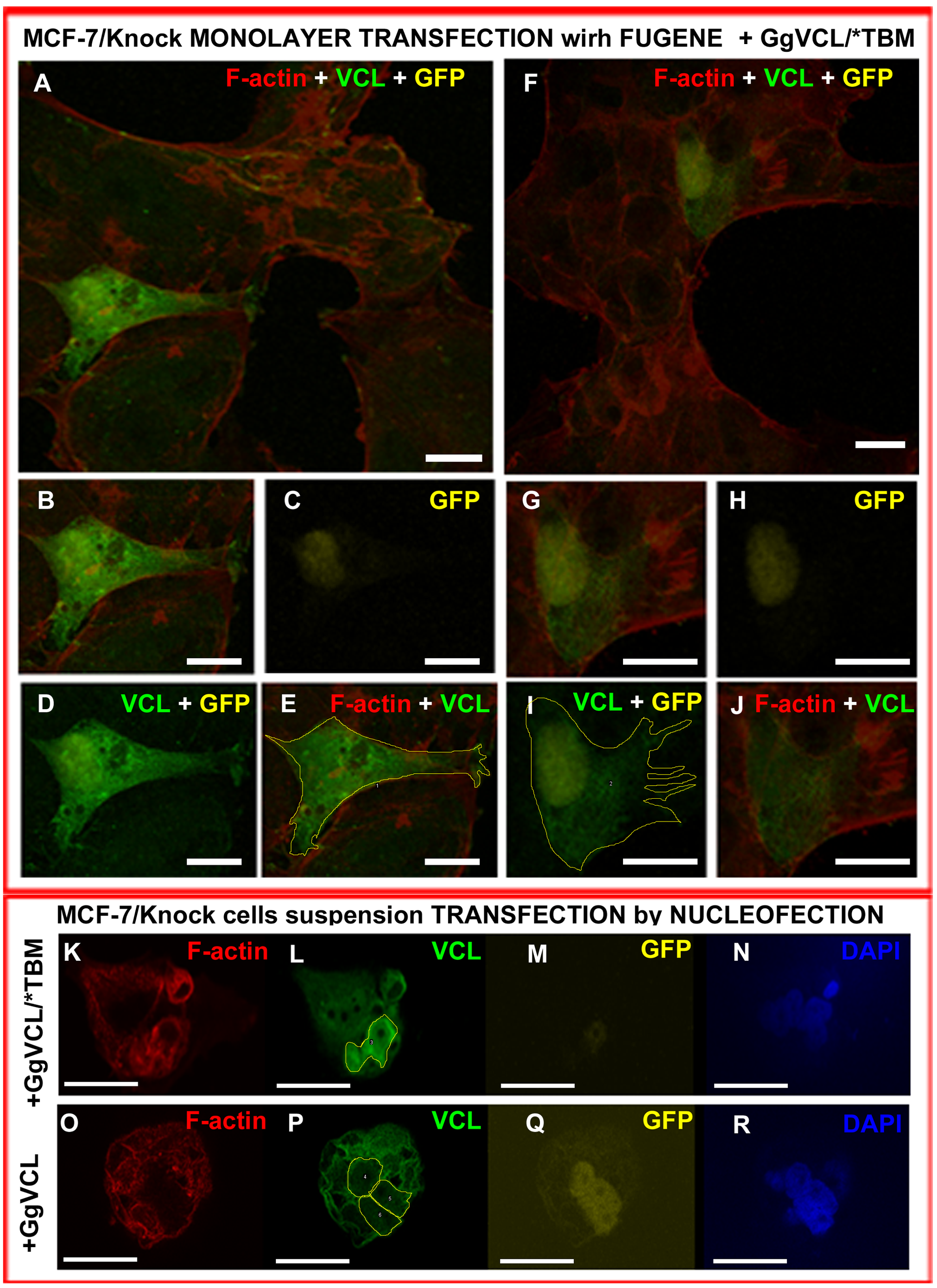

Supplement: Supplemental Information 8 — Cell monolayer was transfected with FUGENE and fixed 48 h later (A-J) or cell suspensions were nucleofected and fixed 20 h later (K-R). GFP (yellow), VCL (green) and F-actin (red) were detected by ICF. (A, F) Monolayer overview, merged channels; (B - E & G-J): enlarged view of two Tol2-VCL/*TBM transfected cells. (B) merge, (C): GFP,(D):VCL+GFP (E): VLC+F-actin+ drawn contour, (G) merge, (H): GFP,(I):VCL+GFP (J): VLC+F-actin+ drawn contour. (K - R) Successful transfection of neighbor cells. (K - N): with Tol2-GgVCL/*TBM; (O - R): with Tol2-GgVCL. (K, O): F-actin, (L, P): VCL, (M,Q): GFP, (N,R): DAPI. Bar: 25 µm [file peerj-09-11442-s008.png]

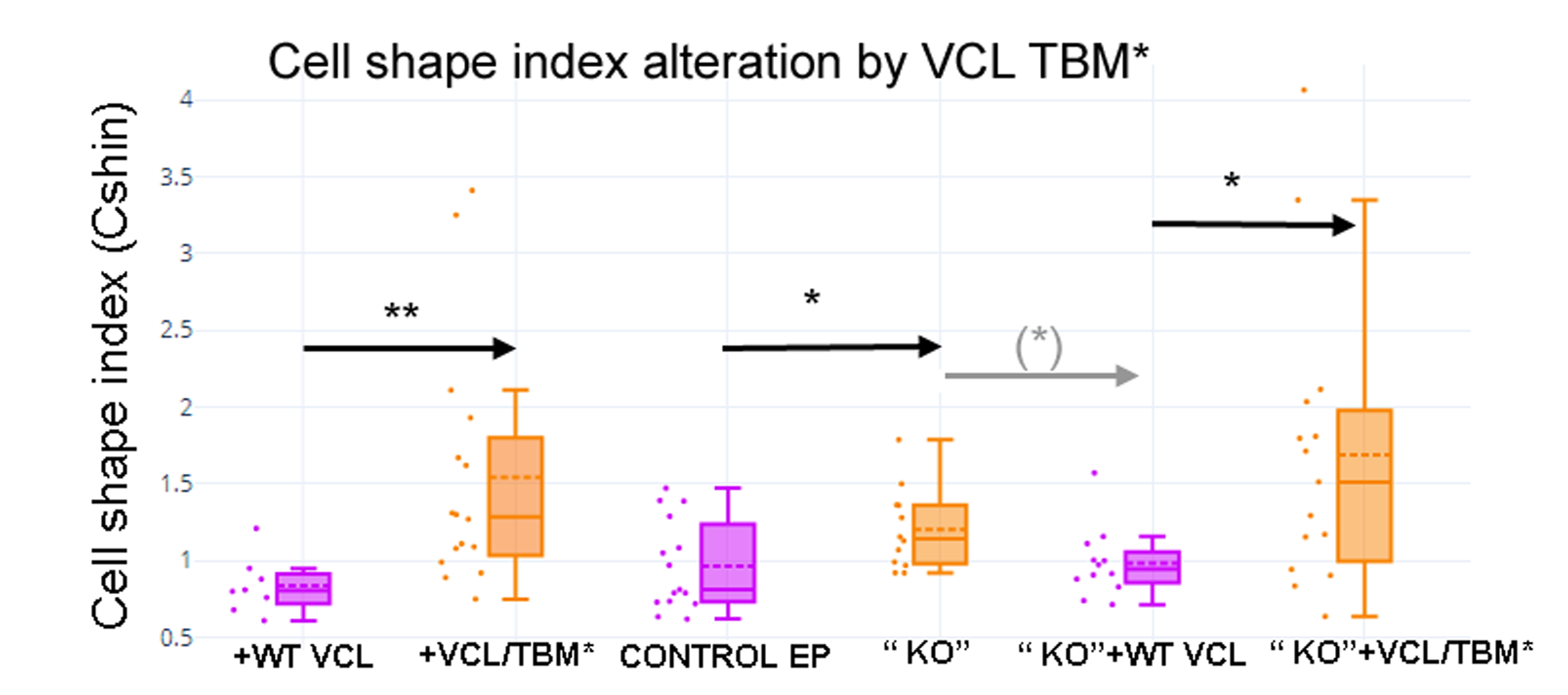

Supplement: Supplemental Information 9 [file peerj-09-11442-s009.png]
